# Disrupted Regional Spontaneous Neural Activity in Mild Cognitive Impairment Patients with Depressive Symptoms: A Resting-State fMRI Study

**DOI:** 10.1155/2019/2981764

**Published:** 2019-01-08

**Authors:** Xiaozheng Liu, Yunhai Tu, Yirou Zang, Aiqin Wu, Zhongwei Guo, Jiawei He

**Affiliations:** ^1^China-USA Neuroimaging Research Institute, Department of Radiology of the Second Affiliated Hospital and Yuying Children's Hospital, Wenzhou Medical University, Wenzhou, Zhejiang 325027, China; ^2^The Eye Hospital, Wenzhou Medical University, Wenzhou, Zhejiang 325027, China; ^3^Tongde Hospital of Zhejiang Province, Hangzhou, Zhejiang 310012, China

## Abstract

Depressive symptoms are common in individuals with mild cognitive impairment (MCI) who have an increased risk of dementia. It is currently unclear whether the pattern of spontaneous brain activity in patients with MCI differs between subjects with and without depressive symptoms. The current study sought to investigate the features of spontaneous brain activity in MCI patients with depressive symptoms (D-MCI) using coherence regional homogeneity (CReHo) analysis with resting-state functional magnetic resonance imaging (rsfMRI). We obtained rsfMRI data in 16 MCI patients with depressive symptoms and 18 nondepressed MCI patients (nD-MCI) using a 3 T scanner. Statistical analyses were performed to determine the regions in which ReHo differed between the two groups in specific frequency bands, slow-4 (0.027–0.073 Hz) and slow-5 (0.010–0.027 Hz), and typical bands (0.01–0.08 Hz). Correlation analyses were performed between the CReHo index of these regions and clinical variables to evaluate the relationship between CReHo and pathophysiological measures in the two groups. Our results showed that D-MCI patients exhibited significantly higher CReHo in the left Heschl's gyrus and left thalamus and lower CReHo in the left postcentral gyrus in the typical frequency band. In the slow-4 frequency band, D-MCI patients showed significantly higher CReHo in the left Heschl's gyrus and left thalamus. In the slow-5 frequency band, D-MCI patients exhibited significantly lower CReHo in the superior medial prefrontal gyrus. In addition, the results revealed that CReHo values in the left thalamus were positively correlated with Hamilton Depression Rating Scale (HAMD) scores in D-MCI patients. These results suggest that the sensorimotor network may be one of the main pathophysiological factors in D-MCI.

## 1. Introduction

Mild cognitive impairment (MCI) is a highly prevalent neuropsychiatric syndrome that often coexists with other disorders in older people [1]. Depressive symptoms are common in individuals with MCI, with a reported prevalence of 32%, and are associated with an increased risk of MCI developing into Alzheimer's disease (AD) [2]. Better insight into the pathogenic mechanisms underlying MCI with depression (D-MCI) is critical for improving clinical intervention and diagnosis.

In recent years, blood oxygenation level-dependent (BOLD) resting-state functional magnetic resonance imaging (rsfMRI), without the requirement of specific experimental tasks, has attracted increasing attention for studying the neural mechanisms of cognitive dysfunction in patients with many psychiatric disorders, such as schizophrenia, depression, and MCI [3–8]. Regional homogeneity (ReHo) analysis has become a popular rsfMRI method and was originally proposed to measure the degree of signal synchronization of fMRI time courses using Kendall's coefficient of concordance (KCC) [3]. ReHo reflects the temporal homogeneity of the regional BOLD signal, and ReHo abnormalities (either an increase or a decrease in ReHo values) are related to unbalanced local brain activity. In recent years, ReHo has successfully been used to investigate brain function in patients with MCI and depression [3–12].

Extensively distributed abnormal brain activity has been observed during the resting state, and associations have been found between some clinical symptoms and specific abnormal patterns of brain activity in patients with depression and MCI [4, 5]. In one previous study, ReHo differences were tested as a differential diagnosis tool in bipolar and unipolar depression [6]. ReHo can be used clinically as a biomarker for the pathophysiology and treatment response of depression [7–9] and for the classification of depression subtypes and MCI [8].

However, a previous study reported that neuronal oscillations are frequency-dependent and that independent frequency bands are generated by distinct oscillators with specific properties and physiological functions [13]. In addition, Liu et al. [14] applied coherence to measure the regional homogeneity (CReHo) or local synchronization of the rsfMRI BOLD signal. These results indicated that CReHo is more sensitive to differences in spontaneous activity than KCC-ReHo between different resting-state conditions (eyes open [EO] vs. eyes closed [EC]).

In the current study, we utilized rsfMRI and CReHo to investigate alterations in spontaneous activity in D-MCI patients compared with nondepressed MCI (nD-MCI) patients. We also investigated the frequency-specific characteristics of CReHo in different frequency bands.

## 2. Materials and Methods

### 2.1. Patients

This study was approved by a Research Ethics Committee of Tongde Hospital of Zhejiang Province, China. All participants (or their legal representatives) gave written informed consent prior to MR scanning. In total, 18 patients with nD-MCI and 16 patients with D-MCI were recruited from July 2013 to August 2016. Each participant underwent a battery of neuropsychological tests, clinical assessments, and neuroimaging examinations and was diagnosed by an experienced psychiatrist.

The criteria for MCI [15] were (a) impaired memory performance, adjusted for age and education; (b) memory complaint lasting ≥6 months; (c) normal general cognitive function (score >24) on the Mini-Mental State Examination (MMSE), as well as the activities of daily living scale (score <26); (d) the clinical dementia rating scale score of 0.5; and (e) the absence of dementia.

Depressive symptoms were identified by professional psychiatrists according to the Diagnostic and Statistical Manual of Mental Disorders, fourth edition [16]. Patients were considered clinically depressed if they exhibited Hamilton Depression Rating Scale (HAMD) scores ≥7 [17] and Neuropsychiatric Inventory (NPI) scores ≥4 [18] in the depression domain [19].

Exclusion criteria were any past or current history of psychiatric disorders, drug or alcohol abuse during the past 5 years, MRI contraindications, or unstable chronic medical conditions.

The local ethics committee approved the study, and all participants provided written informed consent prior to MR scanning.

### 2.2. MRI Scan

MRI data were acquired using a 3.0 Tesla Siemens scanner (Siemens Magnetom Verio; Siemens Medical Systems, Erlangen, Germany). Whole-brain high-resolution T1 structural images with 1 mm isotropic voxels were acquired as a reference for spatial normalization of the data. One 6 min 40 s rsfMRI scan (200 volumes) was acquired with the following parameters: 33 axial slices, thickness/gap = 4.8/0 mm, in-plane resolution = 64 × 64, repetition time (TR) = 2000 ms, echo time (TE) = 30 ms, flip angle = 90°, and field of view (FOV) = 200 × 200 mm^2^. Participants were instructed to lie still and close their eyes during image acquisition.

### 2.3. Data Processing

Data preprocessing was carried out using SPM8 (http://www.fil.ion.ucl.ac.uk/spm) and Resting-State fMRI Data Analysis Toolkit plus (RESTplus) software (http://www.restfmri.net). Detailed preprocessing steps were as follows: exclusion of the first 10 volumes, slice-timing and motion correction, spatial normalization to Montreal Neurological Institute (MNI) space, detrending and temporal filtering with typical temporal bandpass (0.01–0.08 Hz), slow-5 bandpass (0.01–0.027 Hz), and slow-4 bandpass (0.027–0.073 Hz), separately. The data of all participants satisfied the criteria of less than 2 mm of maximal translation of *x*, *y*, or *z* and 2° of maximal rotation.

### 2.4. ReHo Analysis

CReHo analysis was performed using RESTplus (http://www.restfmri.net). Individual CReHo maps were generated by calculating the coherence of the time series of each voxel within its nearest 26 voxels in a voxel-wise analysis. The formula and details of calculating the CReHo value have been described in a previous study [14]. Standardization of CReHo maps was performed by dividing the CReHo of a given voxel by its own mean CReHo within the mask created in the normalization step. The standardized CReHo maps were spatially smoothed with a Gaussian kernel (full width at half maximum (FWHM) = 6 mm).

### 2.5. Statistical Analysis

CReHo differences between the groups were examined in the typical frequency band (0.01–0.08 Hz), slow-5 frequency band (0.01–0.027 Hz), and slow-4 frequency band (0.027–0.073 Hz). A two-sample *t*-test was conducted on the individual normalized CReHo maps in a voxel-by-voxel manner between the D-MCI and nD-MCI groups. To reduce the effect of confounding variables in the statistical analysis, we performed two-sample *t*-tests with the mean relative displacements of head motion, age, and sex as covariates. The resulting statistical map was established using a significance threshold of *p* < 0.05 (AlphaSim corrected for multiple comparisons, with a combined individual voxel *p* value <0.005 with a cluster size >28 voxels). Moreover, with the peak voxels of abnormal regions as spherical centres, spherical ROIs were constructed around these abnormal regions (with a 6 mm radius), and the relationships between mean CReHo values of the spherical ROIs and MMSE, NPI, and HAMD scores for nD-MCI and D-MCI patients were assessed using Pearson's correlation analyses.

## 3. Results

### 3.1. Neuropsychological Results

The nD-MCI and D-MCI groups were well matched in terms of age (*t* = 0.898, *p* = 0.376), sex distribution (*χ*^2^ = 0.161, *p* = 0.735), and years of education (*t* = 0.464, *p* = 0.645). Detailed demographics and the psychological characteristics of the MCI patients are summarized in Table 1.

### 3.2. Abnormal CReHo Values in D-MCI Patients

**Figure 1:**
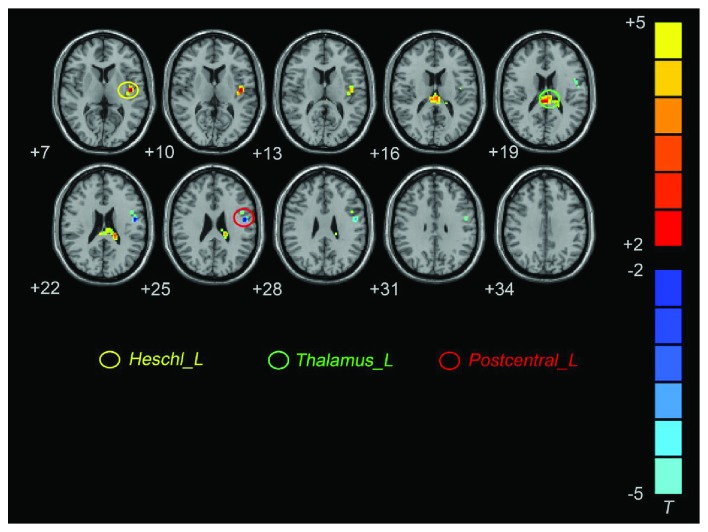
Brain regions showing different CReHo values between the D-MCI and nD-MCI groups in the typical frequency band (contrast = D-MCI − nD-MCI).

**Figure 2:**
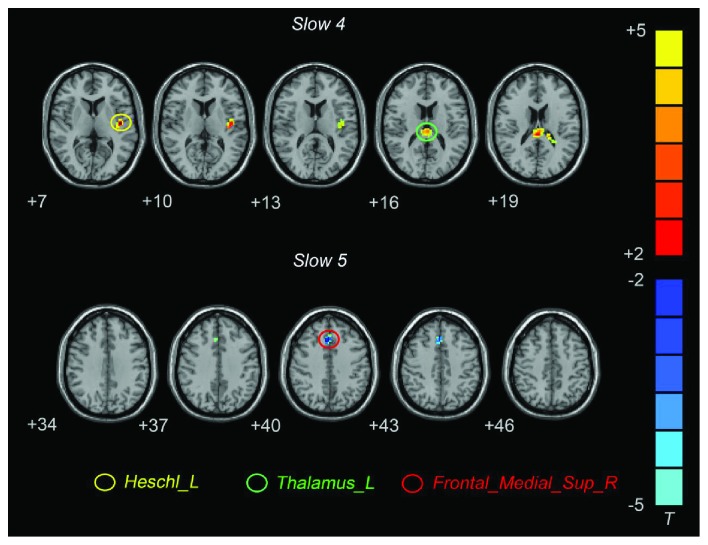
Brain regions showing different CReHo values between the D-MCI and nD-MCI groups in the slow-4 and slow-5 frequency bands (contrast = D-MCI − nD-MCI).

In the typical frequency band, D-MCI patients exhibited significantly higher CReHo in the left Heschl's gyrus and left thalamus, and lower CReHo was observed in the left postcentral gyrus (PoCG) (Figure 1; Table 2). In the slow-4 frequency band, D-MCI patients exhibited significantly higher CReHo in the left Heschl's gyrus and left thalamus (Figure 2; Table 2). In the slow-5 frequency band, D-MCI patients showed significantly lower CReHo in the superior medial prefrontal gyrus (smPFC) (Figure 2; Table 2).

### 3.3. Relationships between CReHo Values and Neuropsychological Data

**Figure 3:**
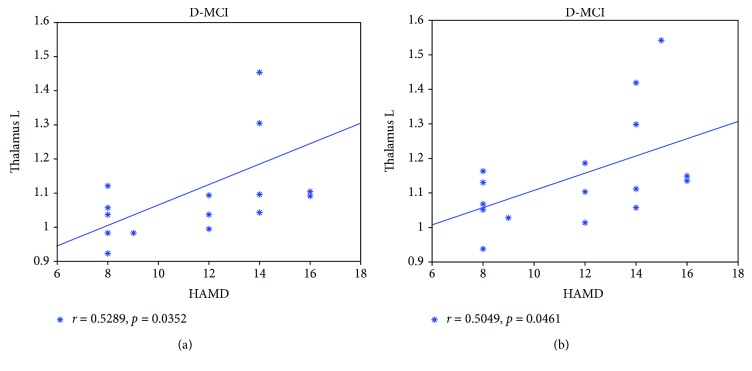
Correlation analysis between the abnormal CReHo values and HAMD scores in D-MCI patients, including the left thalamus in the typical frequency band (a) and the left thalamus in the slow-4 frequency band (b). D-MCI: mild cognitive impairment with depression.

In D-MCI patients, the CReHo values in the left thalamus were positively correlated with HAMD scores in the typical and slow-4 frequency bands (Figure 3).

## 4. Discussion

In the current study, we examined alterations in spontaneous neural activity during the resting state in three different frequency bands (the slow-4, slow-5, and typical bands) in D-MCI patients and nD-MCI patients. In the typical frequency band, D-MCI patients showed significantly higher CReHo than nD-MCI patients in the left Heschl's gyrus and left thalamus and lower CReHo in the left postcentral gyrus. In the slow-4 frequency band, D-MCI patients showed significantly higher CReHo in the left Heschl's gyrus and left thalamus. In the slow-5 frequency band, D-MCI patients showed significantly lower CReHo in the superior medial prefrontal gyrus. In D-MCI patients, the CReHo values in the left thalamus were positively correlated with HAMD scores in the typical and slow-4 frequency bands.

The left PoCG, left Heschl's gyrus, and left thalamus are related to the sensorimotor network. Studies have shown that some patients with MDD suffer from sensorimotor abnormalities, which can manifest as psychomotor agitation or retardation [20]. A frequency-dependent study declared that the topographical balance between the default mode network (DMN) and sensorimotor network (SMN), specifically in the slow-5 frequency band, was significantly increased in depression and positively correlated with clinical scores of depressive symptoms [21]. In patients with bipolar depression, functional connectivity within regions was also reduced in the right and left primary somatosensory areas in the SMN compared with healthy controls [22]. These findings suggested that the SMN is related to the dysregulation of emotion processing in D-MCI patients, which is one of the main pathophysiological factors in D-MCI.

The smPFC is known to be involved in cognitive control [23] and constitutes a core part of the cognitive control network (CCN) [24]. The CCN comprises a set of distinct cognitive domains that include working memory, selective attention, stimulus–response mapping, and performance monitoring [25] and is considered a primary disrupted brain network in MDD. A meta-analysis reported that depressed patients exhibited less connectivity in this network than control subjects [26]. In addition, MDD participants have been reported to exhibit reduced grey matter volume in the dorsolateral and dorsomedial prefrontal cortices [27], and increased depression severity was found to be associated with reductions in medial frontal gyrus volume [28].

Several limitations should be considered when interpreting the current results. First, the relatively small sample size may have affected the statistical power. Second, we were unable to completely exclude the effects of medication on neural activity, potentially limiting the generalizability of our findings. Third, the number of depressive episodes and ratings of standard cognitive scales was not recorded. Thus, we were unable to provide a complete description of all the clinical features and their relationship with brain activity. Finally, this study lacked normal controls. In future studies, comparisons between normal controls and MCI patients should be performed; such comparisons could provide more information to improve understanding regarding the pathophysiology of depression in MCI patients.

## 5. Conclusions

In the current study, we used a CReHo approach derived from rsfMRI to examine the temporal homogeneity of the regional BOLD signal in D-MCI and nD-MCI patients. The results revealed that D-MCI patients, compared with nD-MCI patients, showed abnormal CReHo values, which were mostly located in the SMN. Meanwhile, the CReHo values in the left thalamus of D-MCI patients were positively correlated with HAMD scores. These findings may advance the current understanding of the neurophysiological basis of regional structural-functional specificity in D-MCI patients.

## Figures and Tables

**Table 1:** Demographics and neuropsychological data.

	D-MCI group	nD-MCI group	*t*/*χ*^2^	*p* value
Sex, *n* (M/F)	16 (6/10)	18 (7/11)	0.007	1.000
Age (years)	69.6 ± 6.2	72.1 ± 9.7	0.898	0.376
Education (years)	8.3 ± 2.1	8.5 ± 1.8	0.464	0.645
MMSE	26.6 ± 1.1	26.6 ± 1.0	-0.037	0.971
HAMD	11.7 ± 3.1	0	—	—
D-NPI	7.19 ± 2.3	0	—	—

Data represent the mean ± SD. Data were analysed using independent sample *t*-tests. D-MCI: mild cognitive impairment with depression; nD-MCI: nondepressed mild cognitive impairment; M: male; F: female; MMSE: Mini-Mental State Examination; D-NPI: depression domain of Neuropsychiatric Inventory; HAMD: Hamilton Depression Rating Scale.

**Table 2:** Brain regions with significantly different CReHo values in the D-MCI group compared with the nD-MCI group.

Brain regions	Voxels	BA	MNI coordinates			*T* value
			*x*	*y*	*z*	
Typical frequency band						
Heschl_L	67	48	-45	-12	6	4.5932
Thalamus_L	99		-7	-22	18	3.1034
Postcentral_L	41	48	-51	-6	24	-4.8979
Slow-4						
Heschl_L	52	48	-45	-12	6	4.4832
Thalamus_L	98	21	-7	-22	18	3.4414
Slow-5						
Frontal_Sup_Medial	32	32	3	27	39	-4.7418

D-MCI: mild cognitive impairment with depression; nD-MCI: nondepressed mild cognitive impairment; MNI: Montreal Neurological Institute; BA: Brodmann area.

## Data Availability

The MRI data used to support the findings of this study are available from the corresponding author upon request.
